# Time-bin entangled Bell state generation and tomography on thin-film lithium niobate

**DOI:** 10.1038/s41534-024-00925-7

**Published:** 2024-12-30

**Authors:** Giovanni Finco, Filippo Miserocchi, Andreas Maeder, Jost Kellner, Alessandra Sabatti, Robert J. Chapman, Rachel Grange

**Affiliations:** ETH Zurich, Department of Physics, Institute for Quantum Electronics, Optical Nanomaterial Group, Auguste-Piccard-Hof, 1, 8093 Zurich, Switzerland

**Keywords:** Integrated optics, Quantum information, Qubits

## Abstract

Optical quantum communication technologies are making the prospect of unconditionally secure and efficient information transfer a reality. The possibility of generating and reliably detecting quantum states of light, with the further need of increasing the private data-rate is where most research efforts are focusing. The physical concept of entanglement is a solution guaranteeing the highest degree of security in device-independent schemes, yet its implementation and preservation over long communication links is hard to achieve. Lithium niobate-on-insulator has emerged as a revolutionising platform for high-speed classical telecommunication and is equally suited for quantum information applications owing to the large second-order nonlinearities that can efficiently produce entangled photon pairs. In this work, we generate maximally entangled quantum states in the time-bin basis using lithium niobate-on-insulator photonics at the fibre optics telecommunication wavelength, and reconstruct the density matrix by quantum tomography on a single photonic integrated circuit. We use on-chip periodically-poled lithium niobate as source of entangled qubits with a brightness of 242 MHz/mW and perform quantum tomography with a fidelity of 91.9 ± 1.0 %. Our results, combined with the established large electro-optic bandwidth of lithium niobate, showcase the platform as perfect candidate to realise fibre-coupled, high-speed time-bin quantum communication modules that exploit entanglement to achieve information security.

## Introduction

Quantum information technologies are in need of stable and reliable modules for generation and detection of entangled states^1^. Practical quantum communication schemes make use of the already deployed optical fibre links and have been proved secure to transmit quantum information over more than 500 km^2^. Traditionally, polarisation states of light were exploited, yet random fibre birefringence along communication links renders polarisation encoding impractical to implement on standard telecom networks, therefore spatial mode multiplexing^3^ or time-bin encoding are preferred due to their robustness against polarisation scrambling^4^. Between these two options, time-bin encoding further simplifies the scheme as it can use standard optical fibres instead of multi-core links or sophisticated deconvolution algorithms to distinguish qubits. To streamline the complex task of generating, manipulating and detecting quantum states while maintaining coherence over long communication links, weak laser pulses are typically adopted instead of single photon sources, and decoy states are implemented to enhance security^5–9^ following the BB84 protocol^10^. Among the several proposed approaches for quantum key distribution (QKD), the E91^11^ and BBM92^12^ protocols rely on entanglement to achieve unconditional security. In an entangled QKD scheme, quantum correlations are used to ensure security of the channel and detect eavesdroppers, as a tentative hack of the communication leads to introduction of classical correlations which can be detected by assessing that entanglement has been disrupted instead of implementing more cumbersome bit-error checks as in BB84^10,11^.

As for classical telecommunication, it naturally follows that quantum communication systems also require integrated solutions based on optoelectronic components; these are necessary when aiming at large-scale production and global quantum network deployment^13,14^. Time-bin encoding and Franson interferometry^15^ have been implemented on photonic integrated circuits as part of the efforts to miniaturise communication elements towards fibre-compatible quantum information technologies^16–18^. Among the available platforms able to accomplish the task, the most widespread are silicon-based technologies^3,19–21^, silica^22,23^, or III-V semiconductors; the latter being used as a source of entangled photons^24^ or as pump laser diodes in a hybrid integration scheme^9,25,26^. Vacancy centres or defects in diamond and silicon as well as quantum dots are also being successfully used as sources for applications in quantum information^27–32^.

Lithium niobate-on-insulator (LNOI) is rapidly emerging as a leading alternative for classical communication applications thanks to its low propagation loss, wide transparency range and large electro-optic (EO) bandwidth^33^. Fabrication of high-quality photonic circuits on LNOI is now established and reliable, with low-loss optical circuits being routinely produced for various applications^34,35^. EO modulators have been demonstrated on LNOI to reach several tens of GHz and are now commercially available^36,37^. Ultra-broadband EO-combs^38,39^, generation of short pulses^40^ as well as phase shifters and switches^41^ have also been demonstrated. Additionally, the non-centrosymmetric structure of single crystal lithium niobate films allows for efficient second-order frequency conversion processes by domain engineering and periodic poling^42^. These aspects make LNOI an ideal platform for the next generation of optical communication devices beyond the classical domain, as spontaneous parametric down-conversion (SPDC) can be leveraged to generate entangled photons at the different wavelengths with exceptionally high brightness^43,44^. SPDC is advantageous over spontaneous four-wave mixing (SFWM) not only because of its higher efficiency, but also because the down-converted photons are spectrally well separated from the pump, thus there is no need of high-extinction notch filters required when SFWM is concerned^1^. SPDC in lithium niobate has been widely used to generate highly entangled photon pairs and many experiments used periodically poled bulk crystals, titanium indiffused, proton exchanged or etched waveguides as sources of quantum states for quantum information applications^45–48^. Recent results have also shown that LNOI can be used to perform reliable quantum state generation, qubit control and single photon routing; building blocks for integrated optical quantum computing are thus being developed^49–51^. Furthermore, single-photon detectors integrated on LNOI circuits have been reported^52,53^. As of today, however, there has been no demonstration of generation and reconstruction of time-bin entangled qubits on a single LNOI circuit, which is the natural evolution of a platform aiming at becoming leader of optical communication technologies.

Here, we present an integrated photonic circuit on LNOI that generates time-bin entangled Bell states by SPDC on a periodically-poled lithium-niobate (PPLN) waveguide with 242 MHz/mW on-chip brightness, and performs its tomographic reconstruction with 91.9 ± 1.0% fidelity without background subtraction or active phase stabilisation. We observe quantum interference with 78.1 ± 2.0% visibility, limited only by chromatic dispersion, thus confirming that the generated states are beyond the limit of local hidden variables and can be exploited as a useful resource of quantum information. We observe three orders of magnitude higher source brightness when compared to similar experiments on silicon-based technologies^16,17^.

## Results

### Device, characterisation and experimental procedure

**Fig. 1:**
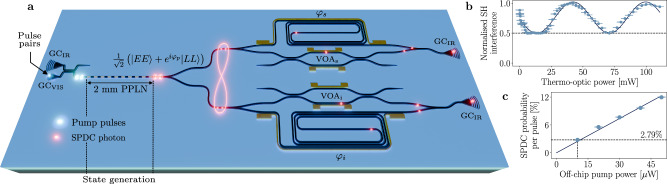
Device, working principle and characterisation. **a** Pump pulses are generated off-chip and input from the left via grating couplers; they undergo SPDC to generate the desired Bell state and probabilistically split to two twin Franson interferometers for tomography. Entanglement is maintained when photons propagate across two analysis interferometers where projection onto the desired bases occurs. GC grating coupler, VOA variable optical attenuator, φ projection phase, s signal, i idler. **b** Normalised second-harmonic interference measured by propagating a 1550 nm femtosecond laser backwards through device and setup. **c** SPDC generation probability versus off-chip average pump power.

The designed optical circuit is illustrated in Fig. 1a. It consists of an optical circuit etched into a 300 nm-thick x-cut magnesium-oxide-doped lithium niobate film. Pulse pairs from an 80 MHz mode-locked laser at 775 nm are generated on the optical table by means of a fibre-based unbalanced interferometer. Before coupling into the device, pulses propagate across approximately 15 m of polarisation-maintaining optical fibre, and are thus stretched by chromatic dispersion from the original transform-limited pulse duration of 100 fs to an estimated of 17 ps. The time-bin delay between two pulses is *τ* ≈ 220 ps to be able to easily resolve all time-bins with our single photon detectors. Pump light is input from the left, coupling in and out of the device is done by using focused grating couplers (GC). A wavelength-division multiplexing device allows to probe the circuit at visible or near infrared wavelengths by selecting the input with appropriate GC. The device consists of two twin Franson interferometers; the long arm is constituted of a delay line matching the pump pulses time separation, while the short arm is equipped with a variable optical attenuator (VOA), realised with a Mach-Zehnder interferometer, tuned by thermo-optic phase shifters (TOPSs)^50^. VOAs are used to compensate for additional propagation losses experienced by the photons travelling on the long arm and maximise interferometric visibility at the recombination point. An additional TOPS running around the delay-line is used to tune the phase of signal and idler photons (*φ*_*s*_, *φ*_*i*_), thus implementing projections of the interfering photons.

Setting up the experiment requires finely tuning the delay of the free space interferometer to match that of the fabricated circuit; this is done by pumping the system in reverse, with a femtosecond laser at 1550 nm which propagates backwards across the delay-line first, and after recombination pulses are fed into the 2-mm-long PPLN for second-harmonic generation (SHG). Simultaneously tuning the VOA to balance the pulses intensity and sweeping the applied voltage to the delay line shows interference of the up-converted signal at the preparation interferometer output. Interference is expected to manifest maximum 50% visibility (four equally intense pulses are present in total, only two of which recombines in time while the other two constitute a constant background), as it is indeed observed and reported in Fig. 1b. Deviations from the sinusoidal behaviour are to be attributed to random phase fluctuations in the table-top interferometer, which is not actively stabilised.

Next, we quantify the pump power required to minimise double down-conversion events following the procedure described in ref. ^54^ on a straight PPLN waveguide fabricated next to the device: the photon generation probability can be estimated by taking the ratio between coincidence counts at zero time delay and at the repetition period of the pump laser. We record coincidences for 60 s over a 30 ns window; the measurement outcome is displayed in Fig. 1c, fitted to a linear model as predicted by theory and shows that, at ${10}\,{\text{uW}}$ of off-chip pump power, the probability of generating a single SPDC pair per pulse is *p* = 2.79 ± 0.09%, thus that of double events is proportional to *p*^2^ ≈ 0.08% and can be neglected. Errors concerning single photon measurements are estimated by assuming Poissonian statistics of the photon counts. Given the GC efficiency of ~ 7 dB and pump pulse duration, we estimate an on-chip average and peak pump power of ${2}\,{\text{uW}}$ and 1.5 mW, respectively. At ${10}\,{\text{uW}}$ off-chip pump power, we measure entangled states at ~ 1800 Hz which, considering the measured Klyshko efficiency^55^ of *η* = −15.5 dB (see Supplementary Material), gives an on-chip source brightness of ~ 242 MHz/mW. The measured values highlight the advantage of using LNOI and SPDC against the more common silicon-based platforms for quantum information applications, as we can achieve similar or higher rates by pumping our source with two to three orders of magnitude lower pump peak power compared to other, fully integrated devices based on SFWM^16,17^.

### Calibration and quantum state description

At sufficiently low pump power, the down-converted photons at the PPLN create an energy-time entangled pair in the form of a Bell state that reads

1 $$\left\vert \psi \right\rangle =\frac{1}{\sqrt{2}}\left(\left\vert EE\right\rangle +{e}^{i{\varphi }_{p}}\left\vert LL\right\rangle \right),$$

where *φ*_*p*_ is the relative phase between pump pulses, control on which allows to generate the maximally entangled $\left\vert {{{\Phi }}}^{\pm }\right\rangle$ states. $\left\vert E\right\rangle$ ($\left\vert L\right\rangle$) describe whether the photon pair has been generated by the leading (trailing) pulse. Due to the type-0 SPDC process, we are forced to probabilistically split photons via Y-junctions; they are then sent to the analysis interferometers. After recombination, photons are out-coupled via infrared GCs and collected by v-groove fibre arrays and guided to commercial superconducting nanowire single-photon detectors; a time-tagger is synchronised to the pump laser clock for counts binning. Further device characterisation is available in the Supplementary Material.

**Fig. 2:**
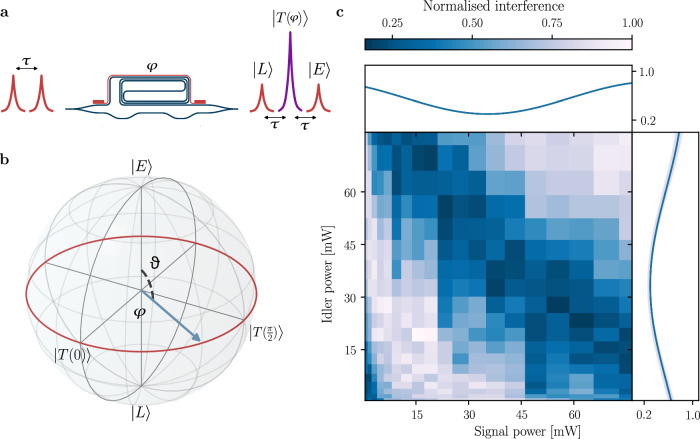
Quantum interference calibration. **a** Schematic of interfering state generation. **b** Single qubit Bloch sphere; the equator can be swept across by tuning the relative phase between interferometer arms. **c** Quantum interference calibration map: oscillation of the quantum state projected onto the energy basis over a wide enough range to span the full Bloch spheres equator. Top/side insets show the interference trend for the signal and idler channels, respectively. Uneven sampling of the map is due to the measurement being taken in voltage steps rather than power.

Generation of the interfering state and its representation on the qubit Bloch sphere are illustrated in Fig. 2a–b. Before entering the analysis interferometer, qubits are in a superposition of $\left\vert E\right\rangle$ and $\left\vert L\right\rangle$ states, which constitute the so-called *time basis* of our experiment and lay at the qubit Bloch sphere poles. After traversing the interferometer, when the early (late) photon has propagated across the long (short) path, an additional state arises as there is no possibility of inferring whether is the early photon to have accumulated a delay or the late photon to have been advanced by a time-bin. This gives rise to quantum interference, which can be controlled by tuning the relative phase between the two interferometer arms. We refer to this additional state as *on-time* state, labelled $\left\vert T(\varphi )\right\rangle$, and its probability amplitude oscillates as a function of *φ* = *φ*_*s*_ + *φ*_*i*_ − *φ*_*p*_. $\left\vert T\right\rangle$ lies on the Bloch sphere equator and specific values of the relative phase allow to build the so-called *energy basis*, which being constituted of non-orthogonal eigenvectors to the time basis allows to perform tomographic reconstruction and to validate the presence of entanglement. After propagation through the interferometer, early and late qubits accumulate twice the delay, we avoid re-defining them for the sake of keeping a light notation. The on-time measurement outcome arise from the probabilistic projection of the time-bin qubit on the Bloch sphere equator.

We calibrate quantum interference by sweeping the electrical power applied to signal and idler TOPSs, and record the oscillation of coincidence counts corresponding to the $\left\vert TT\right\rangle$ state to identify the thermo-optic powers related to different projectors. We perform triple coincidence measurements (signal, idler and pump trigger signal) and collect statistics about photon arrivals in the interfering state. Figure 2c shows a two-dimensional calibration map of signal-idler coincidences with respect to the trigger signal, as a function of signal and idler TOPS powers. Experimental data are fitted to a sinusoidal model and the resulting interference behaviour is shown in the Fig. 2c top/side insets separately for signal and idler channel, respectively. Shaded confidence regions obtained by applying Poissonian statistics are also reported. The map of Fig. 2c covers a wide-enough region to sweep the two projectors across the entire Bloch sphere equator for the two qubits. It serves both to identify the required voltages, but also to extract the pump phase and rotate the reference frame in order to reconstruct a maximally entangled Bell state by the simple transformation ${\tilde{\varphi }}_{s,i}\to {\varphi }_{s,i}-{\varphi }_{p}/2$.

**Fig. 3:**
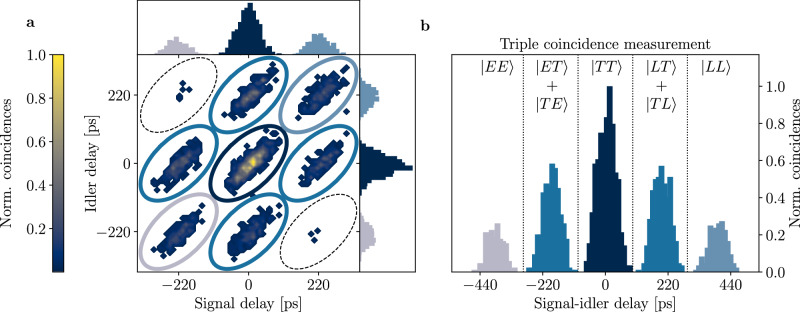
Triple coincidence measurement. **a** Two-dimensional coincidence histogram between signal, idler and pump trigger. Insets show coincidence counts relative to the pump clock on signal and idler channels independently. Drawn ellipses highlight the possible measurement outcomes and are colour-coded correspondingly to the peaks of the collapsed, one-dimensional histogram in (**b**). Labels in panel (**b**) explicitly mark which measurement outcome corresponds to each histogram peak, with the central one representing the interfering on-time state.

Triple coincidence measurements between the common pump clock and photons at the two channels are at the core of our experimental procedure. They enable, thanks to the characteristic feature of Franson interferometry, to simultaneously measure in two non-orthogonal bases, thus the observation of all the possible quantum states originating from probabilistic photon splitting. At the interferometer output and before detection, the quantum state in the joint space, $\left\vert \tilde{\psi }(\varphi )\right\rangle$, features seven possible outcomes (for more information, see Supplementary Material). These can be visualised with a two-dimensional histogram reporting coincidences between signal, idler and pump trigger. This novel measurement technique fully exploits Franson interferometry and increases the amount of retrievable information. An example histogram is illustrated in Fig. 3a, where nine elliptical patterns develop as a function of signal and idler delays, and correspond to each of the possible measurement outcomes. Along the principal diagonal, we find states $\left\vert EE\right\rangle$, $\left\vert TT\right\rangle$ and $\left\vert LL\right\rangle$, which are those of interest for entanglement-based time-bin encoding QKD systems. Off the diagonal are non-interfering states correspondent to distinguishable events to which only early or late photons contribute by taking opposite paths on the two interferometers. The remaining two patterns at the extremes of the anti-diagonal, marked with black dashed ellipses, correspond to outcomes that should not occur if the purest Bell state is produced and we attribute to residual double down-conversion events. Top and side insets show projections of the recorded coincidence on signal and idler channel, respectively, and correspond to the typical measurement that would be performed by each of the two communicating parties. Figure 3b shows a collapsed view of the triple coincidence measurement outcome in one dimension, which features five peaks as reported by other works^24,28^. The drawn ellipses in Fig. 3a are colour-coded correspondingly to the peaks to which counts belong in the collapsed view of Fig. 3b, and labels indicate which specific state falls within each of the histogram sections. It is important to mention that deterministic photon routing would enable the a-priori selection of the quantum state to be observed and eliminate the spurious states belonging to second and fourth histogram peaks, which do not carry useful information. This would allow to optimise data collection and increase coincidence rates depending on whether security of the QKD link is to be confirmed by entanglement quantification or information sharing is desired.

### Quantum state tomography

**Fig. 4:**
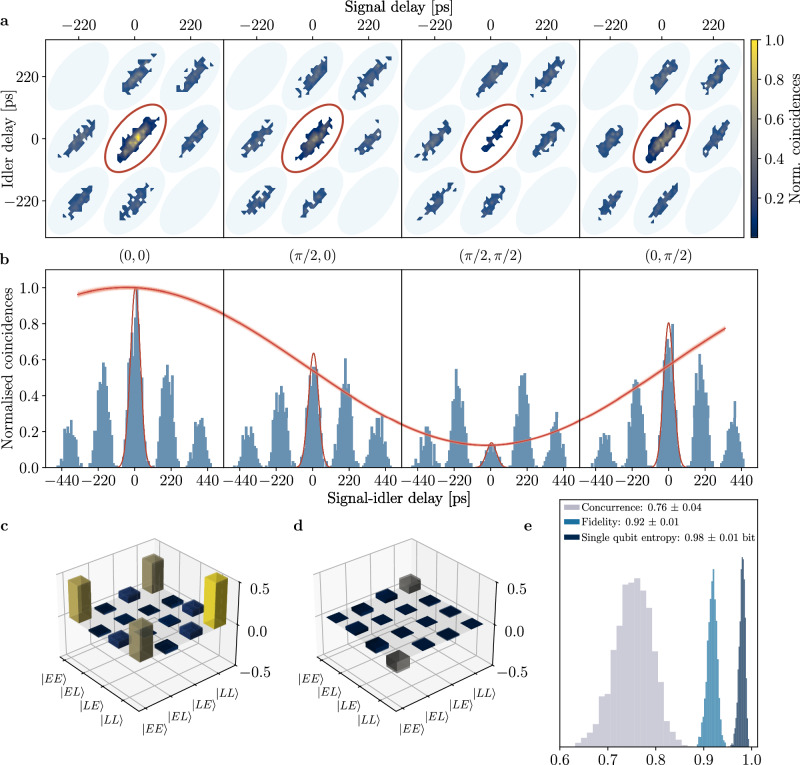
Observed quantum interference and reconstructed density matrix. **a** Two-dimensional histograms correspondent to the four sets of projectors, with the oscillating state marked with a red ellipse. **b** Collapsed triple coincidences with the interfering state fitted to a Gaussian model and the corresponding oscillatory behaviour highlighted. **c**, **d** Tomographic state reconstruction, real and imaginary part, with shaded bars superimposed to the matrices indicating errors obtained via Monte Carlo simulation. **e** Fidelity, concurrence and single qubit entropy histograms originating from the Monte-Carlo simulation, showing high degree of entanglement and suitability of the device for QKD experiments.

Entanglement quantification between the down-converted photons is performed by quantum interference measurement. We set electrical powers on the two interferometers corresponding to projectors at (*φ*_*s*_, *φ*_*i*_) = (0, 0), (*π*/2, 0), (*π*/2, *π*/2) and (0, *π*/2) and record coincidences for 300 s at each point. Counts belonging to the interfering state are observed to oscillate with 78.1 ± 2.0% visibility without need of background subtraction. Figure 4a, b displays the measurement results: two-dimensional histograms (a) and collapsed triple-coincidence measurement (b) for each set of projectors. Counts pertaining to the interfering state are fitted to a Gaussian model, which is integrated over five standard deviations. The integrated counts are used as fitting points to extract the sinusoidal behaviour, with the probability of detecting the interfering state that features the expected oscillation according to

2 $$P=| \langle TT| \tilde{\psi }\rangle {| }^{2}\propto 1+\cos \left({\tilde{\varphi }}_{s}+{\tilde{\varphi }}_{i}\right).$$

We measure an average coincidence rate of 5 Hz, limited by the pump laser repetition rate, additional losses introduced by the time-bin decoding device (probabilistic splitting 3 dB, propagation loss 1 dB, estimated at the VOA output), and the need of further filtering one of the two channels (>10 dB). The broad type-0 SPDC photon bandwidth indeed forces us to place an additional 8.8 nm (full-width at half-maximum) bandpass filter on signal or idler channels, otherwise chromatic dispersion in the waveguide would heavily impact visibility due to the different group velocities of non-degenerate photon pairs. We filter only one channel and rely on energy conservation to post-select photons within a narrower spectral range in order to increase visibility. We measure an interference visibility at the limit of what the filtered photon bandwidth allows; an extended discussion on effects impacting visibility is available in the Supplementary Material. Photon bandwidth and deterministic splitting can be simultaneously achieved by adopting type-II^56^ or counter-propagating^57^ SPDC. These approaches would immediately yield more than 13 dB increase in coincidence counts without changing experimental setup or procedure, while additional 11 dB could be gained by increasing the pump repetition rate to 1 GHz. Importantly, the average pump power used in this experiment is highly conservative as it corresponds to a probability of double down-conversions lower than 0.1 %. We chose this regime in order to avoid further limiting factors to the visibility other than chromatic dispersion. The pump power could easily be doubled with a corresponding two-fold increase in photon counts with better engineered dispersion or photon bandwidth.

We then perform quantum state tomography of the generated qubits following the procedure described in refs. ^58,59^. Maximum likelihood estimation allows us to reconstruct the quantum state with 91.9 ± 1.0% fidelity to a Bell state $\left\vert {{{\Phi }}}^{+}\right\rangle$. Real and imaginary parts of the density matrix are displayed in Fig. 4c, d, with errors being illustrated by shaded bars on top of the reconstructed matrix and calculated by running five thousand iterations of a Monte-Carlo simulation assuming Poissonian statistics of photon counts. We observe a residual phase in the reconstructed matrix, which we attribute to pump phase fluctuations in the preparation interferometer that is not actively stabilised. This nevertheless shows that our calibration procedure is robust against small phase fluctuations and allows us to immediately set the desired projection angles. Figure 4e shows histograms of the calculated relevant quantities on the reconstructed quantum state obtained via Monte-Carlo simulation. Other than the fidelity, we report a concurrence of 0.76 ± 0.04, which is enough to guarantee violation of the Clauser-Horne-Shimony-Holt inequality and further proves the presence of highly entangled qubits^60,61^. We quantify the von Neumann entropy of the generated state to 0.53 ± 0.05 bit, with the theoretical limit being 0 bit. We attribute deviations from the expected value to phase fluctuations at the pump preparation interferometer. Nevertheless, by tracing out one qubit, we quantify the von Neumann entropy in the reduced space to be 0.98 ${\pm}$ 0.01 bit. This shows that separation of the two qubits results in a close to completely mixed state, hence further confirming that a highly entangled state was generated. These results confirm the suitability of our platform as transmission and reception modules of quantum information for entangled time-bin encoding QKD protocols.

**Fig. 5:**
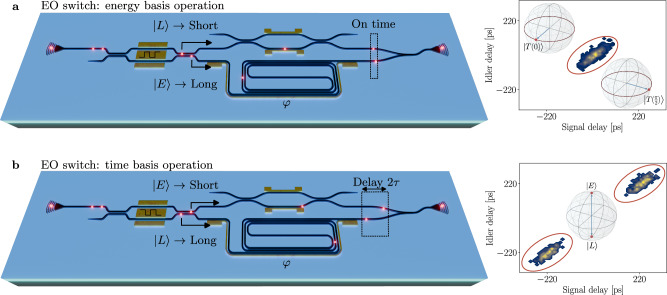
Proposed QKD receiver device. Electro-optic modulated Franson interferometer for deterministic photon routing. **a** Energy basis regime to maximise counts flowing into the interfering state and confirm channel security. **b** Time basis regime to deterministically split qubits in time and use them as source of secure quantum information. Example two dimensional histograms and state representation in the Bloch sphere are reported on the side panels for the two cases.

Last, we propose a modification to our device towards an implementation as QKD receiving module. Addition of high-speed EO switches would enable to route early and late photons to the desired interferometer arm for projection in the time or energy basis, as illustrated in Fig. 5. In the prospect of employing the device for QKD demonstrations, the operation regime would be alternated in order to periodically confirm the presence of entanglement by directing all photons to the interfering state ($\left\vert E\right\rangle$ to the long arm and $\left\vert L\right\rangle$ to the short arm) (Fig. 5a) in order to maximise counts in the central peak and rapidly confirm security of the link. Once entanglement is confirmed, the operation can be reversed in order to temporally split the photons which would then serve as unconditionally secure source of information by leveraging entanglement (Fig. 5b). The side panels display the corresponding pattern that would develop on the two-dimensional histogram for the two operation regimes, and projected states are highlighted in the accompanying Bloch spheres. One option to realise QKD modules would be to have a first device on Alice’s side performing state preparation and projection measurement of one qubit. The second qubit would be sent to Bob’s receiver chip on the opposite side of the link, and he would then perform projection measurements on it. The communication scheme would then follow the typical procedure of E91 or BBM92 protocols. Incidentally, with the described conditions, state preparation could also be performed by a third party, Charlie, who would distribute the two qubits to the communicating parties in a semi-device-independent scheme.

## Discussion

Integrated photonic circuits are a promising solution to the long sought implementation of large-scale, unconditionally secure quantum communication networks. In this work, we showed that lithium niobate-on-insulator, that has been already proved leading platform for classical telecommunication, can be exploited also in the quantum regime. Our results show that thin-film lithium niobate outperforms silicon-based technologies in the generation and reconstruction of entangled quantum states in terms of state generation efficiency, thus required pump energy to achieve similar or higher single photon count rates. By combining our results with the already available high-speed switches and integrated single photon detectors, there is clear potential for the development of reliable transmitters and receivers of quantum information on a single platform to enhance communication security using standard telecommunication networks. While at the current stage information rates are still limited by the pump laser repetition and the need of photon filtering, pulses can be carved out of continuous wave laser diodes, heterogeneously integrated on the platform^26^, in order to boost the data-rate. We chose 220 ps-wide bins due to a conservative approach, but the limiting factor of our system is only its timing jitter, which corresponds to approximately 50 ps. We estimate that pulses could be carved at 10 GHz, which corresponds to 100 ps pulse pairs at a 5 GHz rate. Under these conditions, by employing deterministic SPDC photon separation, reducing their bandwidth or engineering waveguide dispersion would yield more than 40 dB in coincidence count-rates (18 dB for increased pump rate, 3 dB for double pump power, 10 dB for removing the need of filtering, 8 dB for EO modulation, 3 dB for deterministic separation of SPDC). Additionally, lower losses between circuit and detection would also translate into an increase in count-rates, with the ultimate goal being integration of single photon detectors on chip. Moreover, the full bandwidth can be exploited to wavelength-multiplex the communication and further increase channel capacity^48,62^ towards MHz-rate entangled quantum key distribution. Our results confirm that lithium niobate-on-insulator is a promising platform for energy-saving solutions to the quest of realising integrated communication modules for large scale quantum information networks towards unconditionally secure data sharing.

## Methods

### Design and fabrication

Optical circuits and delay lines are simulated using finite element (COMSOL Multiphysics), finite difference eigenmode simulations (Ansys Lumerical) and custom scripts to extract chromatic dispersion in the long spirals by taking into account anisotropy of the crystal (see Supplementary Material). A waveguide top-width of 1200 nm is chosen together with an etch depth of 200 nm on a 300 nm-thick, 5 % MgO-doped, x-cut lithium niobate-on-insulator substrates with 2-μm-thick buried oxide layer on a silicon handle. The waveguides support optical modes at both 775 and 1550 nm, and we choose such a cross-section in order to enable efficient frequency conversion between the two waves^63^. The performance of the circuit at 775 nm is of no concern after down-conversion has happened in the first 2 mm of propagation, as the pump photons are filtered out before detection. At 1550 nm, the fundamental transverse-electric (TE) mode, TE_01_, is well confined (effective index ${n}_{eff,T{E}_{01}}=$ 1.67) while the TE_02_ and the first order transverse-magnetic (TM_01_) modes are very weakly supported (${n}_{eff,T{E}_{02}}=$ 1.51 and ${n}_{eff,T{M}_{01}}=$ 1.46, respectively). We use highly polarisation-selective grating couplers, thus coupling into the TM mode is very unlikely, especially seen the fact that type-0 phase matching implies that the down-converted photons share the same polarisation as the pump. Coupling to the TE_02_ mode, instead, is very weak due to a poor overlap with the fundamental mode given their symmetries. Power transfer between them would require tight waveguide bends with a radius lower than 20 μm. We use bending radii of 100 μm to avoid mode crossing and employ Euler bends in order to minimise loss and dispersion^64^.

VOAs and TOPSs are designed by combining optical finite element with heat transfer simulations and a coupled-mode theory approach as in ref. ^50^.

First, comb-shaped electrodes for periodic poling are deposited by patterning with electron-beam lithography (EBL) a double-layer polymethyl methacrylate (PMMA) resist, followed by lift-off of 100 nm of chromium. The crystal is poled by applying a train of high-voltage pulses and poling quality is ensured by non-invasive two-photon microscope imaging. An array of PPLN regions is patterned on the sample at first, and after imaging the layout is adapted to connect those best matching the design parameters to the Franson interferometers for the next lithography step. Waveguides are defined by EBL on a 500-nm-thick hydrogen silsesquioxane resist and the film is etched 200 nm by Argon ion milling. Redeposited material is removed by wet cleaning using a potassium hydroxide solution following the process described in ref. ^35^. TOPSs are patterned with an additional EBL step and lift-off process of 100 nm of gold with 5 nm of chromium adhesion layer. Electrodes are routed to the edge of the chip by means of a second gold layer patterned by direct laser writing and lift-off. Finally, electrodes are wire-bonded to a printed circuit board for simultaneous control of all heaters via software.

### Measurements and data analysis

A Ti:Sapphire mode-locked laser at 80 MHz provides pump pulses for SPDC. The laser is coupled into polarisation-maintaining fibre and guided to a fibre-based table-top unbalanced interferometer where pulse pairs are produced. Pulses propagate for approximately 15 m of fibres, which according to the manufacturer’s data manifest a chromatic dispersion parameter of roughly *D* = −130 ps/nm/km at the pump wavelength. Given the pump bandwidth of 8.8 nm at the source, we calculate a temporal broadening of ~ 17 ps before coupling into the chip. Light is coupled in and out of the devices by using v-groove fibre arrays through focused grating couplers etched into the LN film. Classical characterisation is possible by either pumping the system in reverse and inspecting the generated second harmonic signal from a femtosecond telecom laser, or forward by using a wavelength division multiplexing device that allows to couple 1550 nm light into the circuit by evanescent field coupling. Additional GCs at all ports of the Mach-Zehnder interferometers (not illustrated in the circuit schematic) allow to cross-check the device behaviour at all configurations of input/output. Custom python software, a voltage source and custom electrical circuitries allow to automatise all classical and quantum measurements. VOAs are characterised by monitoring the additional ports and 1550 nm pulses are measured with a fast photodiode and oscilloscope while the tuning voltage is swept until the balance point is found. Balancing the interferometers can also be done by monitoring single photon coincidences and making sure that histograms corresponding to $\left\vert E\right\rangle \,\left\vert L\right\rangle$ states have equal amplitude. Time-stamping single photon counts is done by synchronising our time-tagger device with an electrical trigger signal extracted from the pump laser cavity. For single photon measurements, device outputs are filtered with long-pass filters (>60 dB extinction) on both channels to remove residual pump photons that would saturate the detectors or add deleterious background. An additional bandpass filter on the idler channel allows to post-select photons within a narrow spectral region for enhanced interference visibility.

Data is analysed in Python with custom software partially based on the QuTip package.

## Supplementary information

Supplementary Material: Time-bin entangled Bell state generation and tomography on thin-film lithium niobate

## Data Availability

Data supporting the findings of this study are available within the article and Supplementary Material. Raw data is available from the corresponding author upon request.

## Code availability

Codes to analyse the data and produce figures are available from the corresponding author upon request.
