# Supplementary material for: Time-bin entangled Bell state generation and tomography on thin-film lithium niobate
Source: npj Quantum Inf. 2024 Dec 30;10(1):135. doi: 10.1038/s41534-024-00925-7 (PMC11685101; doi:10.1038/s41534-024-00925-7)
Supplement: Supplementary file 1 — Supplementary Material: Time-bin entangled Bell state generation and tomography on thin-film lithium niobate [file 41534_2024_925_MOESM1_ESM.pdf]

Supplementary Material: Time-bin entangled Bell state generation and tomography on thin-film lithium niobate

Giovanni Finco,* Filippo Miserocchi, Andreas Maeder, Jost Kellner,
Alessandra Sabatti, Robert J. Chapman, and Rachel Grange
ETH Zurich, Department of Physics,
Institute for Quantum Electronics, Optical Nanomaterial Group,
Auguste-Piccard-Hof, 1, 8093, Zurich, Switzerland

(Dated: December 3, 2024)

- **SM1: Setup and characterisation**
- **SM2: Device calibration**
- **SM3: Effects impacting visibility**

* gfinco@phys.ethz.ch

SM1: SETUP AND CHARACTERISATION

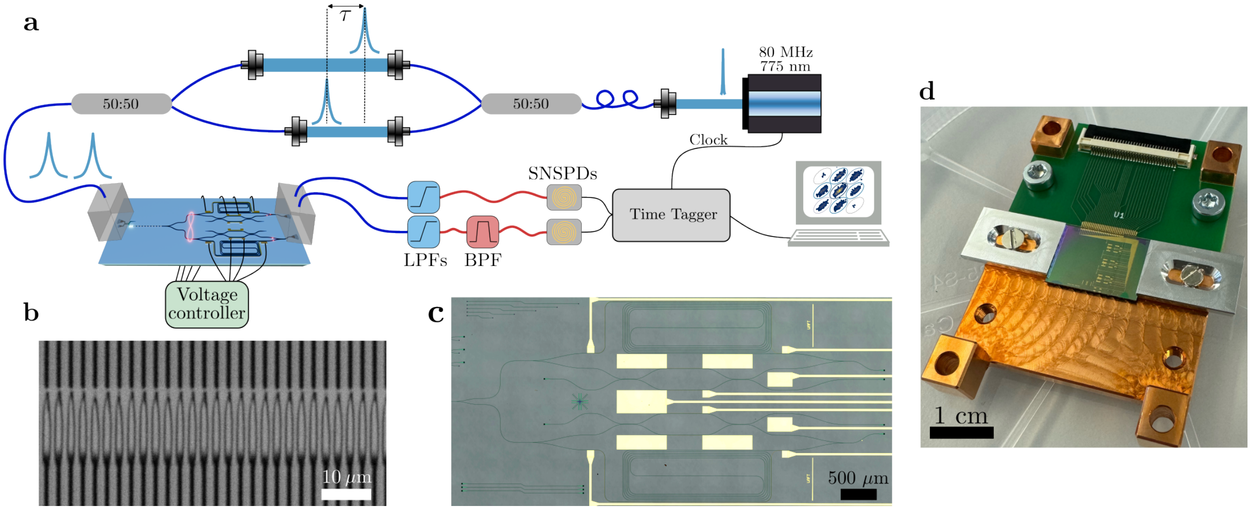

Figure SF1. **Experimental setup and device.** **a** Schematic of the experimental setup. LPF: long-pass filter, BPF: bandpass filter, SNSPD: superconducting nanowire single photon detector. **b** Two-photon microscope image of a fraction of poled waveguide, dark lines are the electrodes fingers. **c** Optical microscope image of a fabricated device, excluding input and poled waveguide. **d** Image of the final sample, including holder and printed circuit board.

Figure SF1a illustrates a schematic of the experimental setup: an 80 MHz pump laser at 775 nm with 100 fs transform limited pulse duration is coupled into polarisation maintaining fibres and pulse pairs are generated with an unbalanced table-top interferometer. After propagating through approximately 15 m of polarisation maintaining optical fibre, pulses are stretched to an estimated duration of 17 ps given the dispersion parameter of $D = -130$ ps/nm/km at 775 nm. Pulses and output photons are coupled in and out of the chip via v-groove fibre arrays and focused grating couplers. Two long-pass filters suppress the residual pump power and a bandpass filter on one of the two channels post-selects SPDC photons within a spectral range that allows to observe large interferometric visibility. Photon are detected with superconducting nanowire single photon detectors (SNSPDs) and time-binning is performed with a time-tagger system, synchronised with an electronic trigger signal from the pump laser. Figure SF1b display a two-photon microscope image of a poled film region before patterning and etching of the waveguides. Figure SF1c-d show an optical microscope picture of the device, excluding inputs and poled waveguide and a picture of the

completed sample (which includes three copies of the device), respectively.

Figure SF2 reports examples measured transmission spectra of focused grating couplers operating at 775 nm (a) and 1550 nm (b). The measurements were obtained from short test

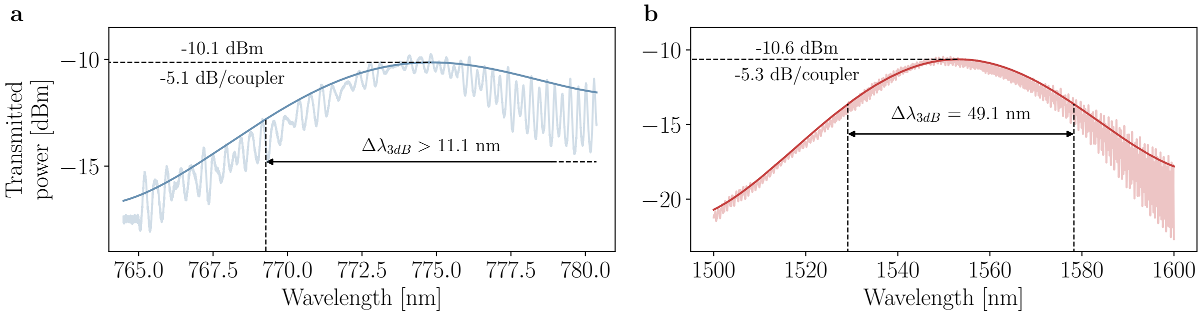

Figure SF2. **Grating couplers characterisation.** Transmission spectrum of test grating couplers operating at **a** 775 nm and **b** 1550 nm. The shaded lines are actual transmission spectra affected by Fabry-Perot interference between input/output interfaces, while the solid line is the smoothened spectrum. Maximum transmission and 3 dB-bandwidth are indicated within the plot. Measurements were obtained by sweeping continuous-wave lasers with 1 mW off-chip power over the available bandwidth.

waveguides fabricated on the same sample, with an off-chip continuous-wave laser power of 1 mW, swept across the tunable bandwidth. The maximum transmitted power is thus to be interpreted as (twice of) the coupling efficiency of each grating coupler since both input and output ports influence the measurements. We measure coupling efficiencies in the order of $\sim$−5 dB per grating at both wavelengths, and a 3 dB-bandwidth of >11.1 nm and 49.1 nm, respectively for visible and near-infrared wavelengths. These results are in line with our technological standards on monolithic structures produced with a single lithography and etching step, and confirm that we can efficiently couple both the pump signal in and the down-converted photons out of the device. Important is to mention that these measurements are obtained by coupling light in and out of the chip using single-mode fibres, which are of easier alignment. During quantum experiments, we used fibre-arrays in order to collect both outputs, and the difficulty of perfectly aligning those introduces additional coupling losses, leading us to an estimate of approximately 7 dB of coupling loss per grating. Interference fringes in the transmission spectra are Fabry-Perot resonances between input and output ports as discussed below; the measured spectra are shown with shaded lines while the solid

curves are obtained by filtering the oscillations out.

Figure SF3 shows the transmission spectrum of an unbalanced interferometer over 100 nm bandwidth centred at 1550 nm, obtained by sweeping a CW laser across the bandwidth of interest. While additional interference patterns arise due to the interferometer itself and the presence of further optical interfaces and small scattering centres, the maximum transmission only slightly varies across the observed photon bandwidth during the experiments as can be seen from the zoom-in in Fig. SF3b. We adopt highly broadband devices when designing our circuits in order not to face further bandwidth limitations: power splitting between the interferometer arms is done by using Y-splitters, while the transmission spectrum of the variable optical attenuators qualitatively coincides with that of the grating couplers.

The free spectral range of an unbalanced Mach-Zehnder interferometer is calculated as

$$FSR = \frac{\lambda^2}{n_g \Delta L}, \tag{1}$$

where for our design the difference in arm length equals $\Delta L = 29.81$ mm and the group index is again assumed from simulations to be $n_g = 2.19$ at $\lambda = 1550$ nm (see end of the section). With these parameters, we estimate an FSR of 36.8 pm, and we measure a very consistent value of 36.5 pm as illustrated in the inset of Fig. SF3a (zoom-in of a 0.24 nm around 1550 nm), thus confirming the quality of our design procedure.

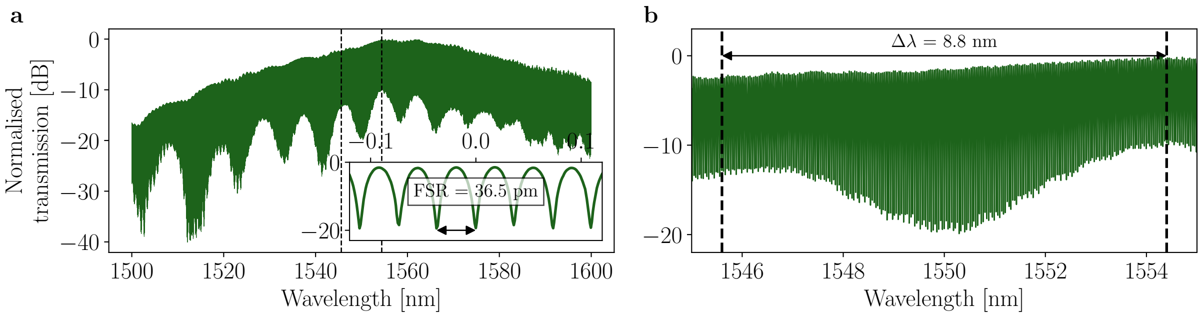

Figure SF3. **Unbalanced Mach-Zehnder interferometer transmission spectrum.** Obtained by coupling the around 1550 nm over **a** 100 nm bandwidth and **b** close-up over the bandwidth of interest for the experiments. The inset shows a zoom-in of a 0.24 nm-wide region around 1550 nm where we highlight the average free-spectral range of the device.

As quantum measurements are performed in coincidence, and the collected counts average over all combinations of photon wavelengths that satisfy energy and phase-matching

conditions, interference fringes are averaged out. Furthermore, interference is observable using a broadband signal only when two interferometers are combined (preparation of pulse pairs and analysis device). This is because, during preparation, a relative phase is imparted between two subsequent pulses, which sets a phase relationship between all wavelengths participating to the interference when photons are recombined, and is compensated for by the projection interferometer. Importantly, quantum measurements make interference visible by time-binning of the photon counts, while with classical pulses we can only observe an average effect.

We characterise source brightness and system loss by measuring the Klyshko efficiency [1] on a straight waveguide fabricated next to the test device for time-bin experiments. The waveguide is pumped with varying average power, the output is collected and filtered to suppress the residual pump photons before being probabilistically split with a 50:50 fibre splitter. The two channels are monitored using SNSPDs to record both single and coincidence counts. The measured values are reported in Fig. SF4, where the top panel shows single counts (for both channels) and coincidences versus off-chip pump power. Average

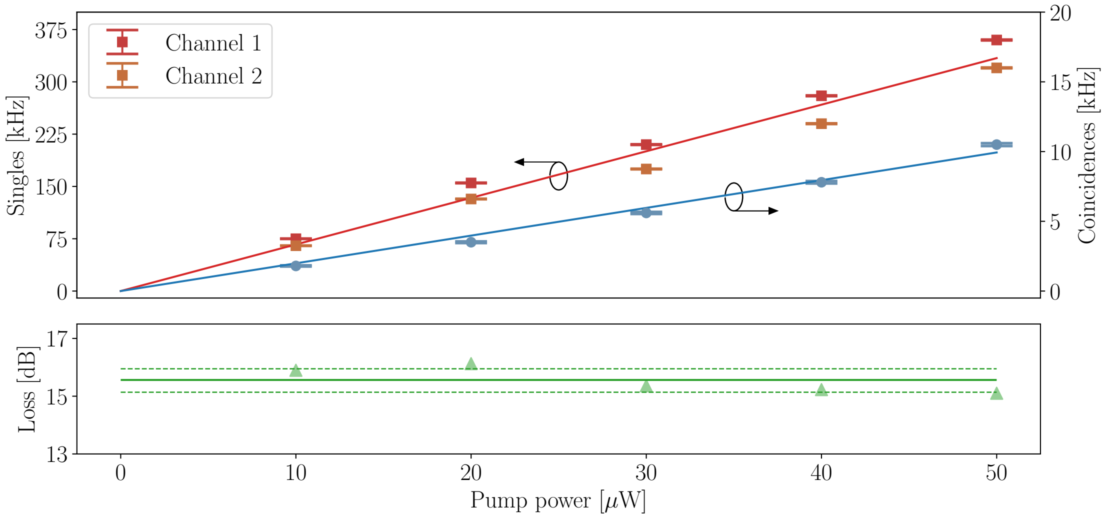

Figure SF4. **Total system loss.** The top panel shows single counts at two SNSPDs channels and the measured coincidences along with linear fits to the data. Counts are generated and collected from a straight waveguide fabricated next to the device. The bottom panel reports the system loss versus off-chip pump power, with its average and standard deviation marked with horizontal solid and dashed lines, respectively.

singles and coincidences are fitted to a linear model as theory predicts. The bottom panel shows the system loss, calculated as the ratio between singles and coincidences, with the solid and dashed lines representing average loss and standard deviation, respectively. We measure 15.5 dB of average loss which gives, considering the grating coupler efficiency of ∼7 dB, an on-chip source brightness of ∼242 MHz/mW. Additional 7 dB loss characterise the time-bin device due to probabilistic photon splitting and propagation loss in the waveguides.

We follow the procedure described in Ref. [2] to quantify the relationship between pump power and SPDC probability per pulse. Figure SF5 displays the results and extends the data reported in the main manuscript. By calculating the ratio between coincidence peaks

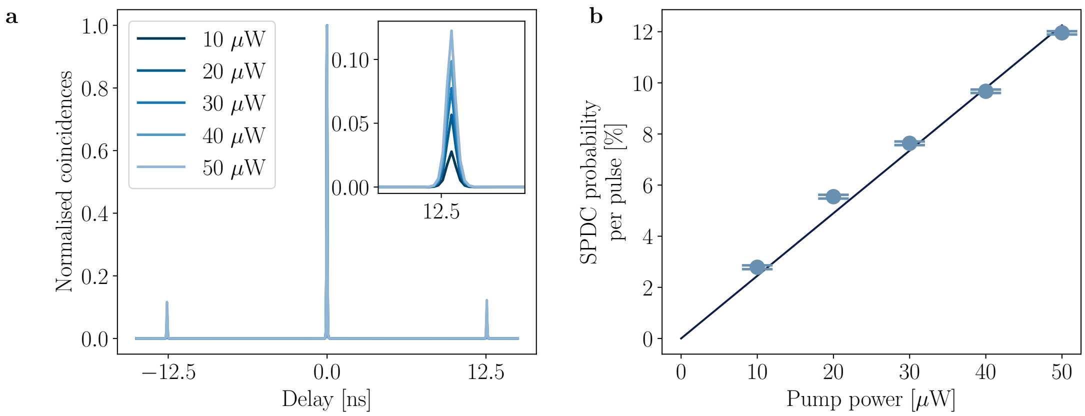

Figure SF5. **Extended Figure 1(c): Spontaneous parametric down-conversion probability.** **a** Normalised coincidences over a time interval covering two pump periods, with the inset illustrates the secondary peak and its decrease in relative amplitude as the pump power is lowered. **b** SPDC generation probability per pulse versus off-chip pump power.

at zero time-delay at the repetition period of the pump laser, one can extract the probability of generating a photon pair from a pump pulse under the assumption of large enough system loss, which applies in our case. The workflow is based on the argument that if a photon from a pair is lost before detection, it may coincide with one originating from the next pulse in the sequence; this event is progressively more likely as the pump power is increased as the number of double down-conversion events is enhanced. Figure SF5a shows normalised coincidence counts over a time interval covering two pump periods, while the insets highlights the relative decrease of the secondary peak as the pump power is lowered, indicating a

decreased probability of photon pair generation. SPDC generation probability per pulse versus off-chip pump power is reported again in Fig. SF5b

Figure SF6 reports spectral characterisation data of our periodic poling. Figure SF6a displays the second harmonic generation (SHG) spectrum (solid line) and measured coincidence counts (data-points, interpolated without specific functional form) for a straight waveguide fabricated next to a time-bin encoding device. The SHG spectrum is obtained by sweeping a continuous-wave infrared laser and recording the output SH power. Interference fringes originate from Fabry-Perot interference between small reflections at the input and output grating couplers. The relatively broad conversion bandwidth and skewed overall shape are due to the limited poled region length and thickness variations across the lithium niobate film, which shift the phase matching wavelength. Coincidence counts are recorded by tuning the central wavelength of the pulsed pump laser across the SH bandwidth. Although the pump laser spectrum (approximately 9 nm-wide, qualitatively displayed with a thin solid line in the background when centred at 775 nm) largely overlaps with the full SH bandwidth, we observe a consistent increase of coincidence counts as the central wavelength is swept across it in correspondence of the most efficient SH generation. While the SH is obtained by sweeping a continuous-wave laser with kHz bandwidth across the phase matching window, and thus the SH spectrum has pm resolution, the discrete points of coincidence counts correspond to the central wavelength of the pulsed laser used as pump. Consequently, the coincidence data do not correspond to (half of) the wavelength of the down-converted photons, but to the wavelength of the pump photons they match energy and momentum with. Indeed, each data point is to be seen as an average of all photon pairs generated by pump photons from a pulse centred at the indicated wavelength, which can be highly non-degenerate. For this reason, any interference effect is averaged out and the data points are distributed on a smooth curve. This also reflects on the observed visibility, as we only measure interference originating from the ensemble of photons which are energy and momentum matching within the filtered bandwidth.

Figure SF6b illustrates an inferred difference-frequency generation (DFG) map. We conduct sum frequency generation measurements by sweeping two continuous-wave telecom lasers across the phase matching bandwidth and reconstruct the expected DFG map from it by energy conservation arguments. The results highlight the broad phase matching bandwidth of more than 50 nm by relating pump wavelength on the x-axis and idler (or signal)

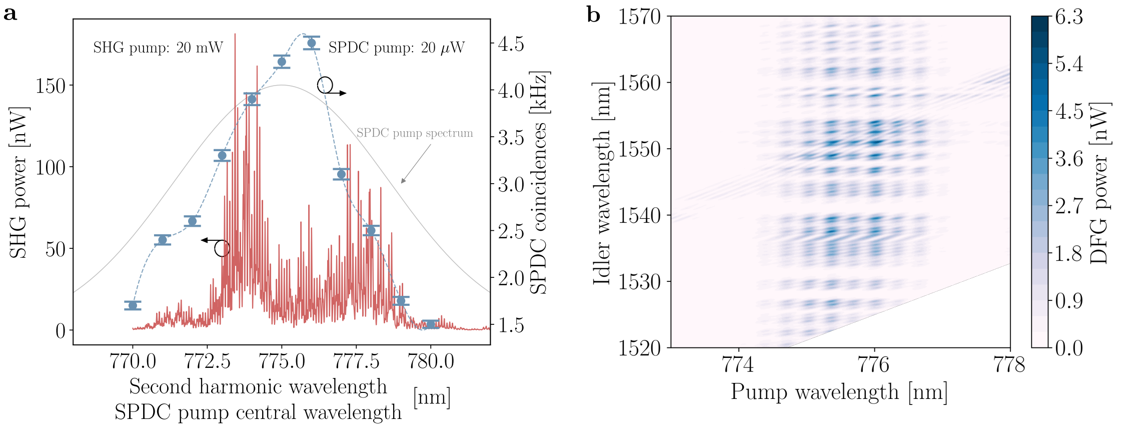

Figure SF6. **Characterisation of periodically poled waveguides.** **a** Second harmonic generation spectrum and measured coincidence counts as a function of the pump wavelength. The thin solid envelope qualitatively illustrates the spectral width of the pump laser when centred at 775 nm. **b** Inferred difference frequency generation map illustrating the broad phase matching bandwidth of spontaneous parametric down-conversion in our waveguides.

wavelengths on the y-axis. This confirms that the generated SPDC photons can be highly non-degenerate and forces us to utilise a bandpass filter to post-select photons within a narrower spectral range in order to enhance visibility as already discussed.

Close inspection of the second harmonic spectrum reveals two patterns indicating the presence of resonances in the waveguide. Figure SF7a shows a detail of the SH spectrum at 775 nm. By considering the free spectral range (FSR) between neighbouring peaks of high and low intensity, respectively labelled FSR_1 and FSR_2, we calculate them to be on average 0.09 nm and 0.08 nm. The free spectral range for an optical mode in a Fabry-Perot cavity is calculated as

$$FSR = \frac{\lambda^2}{2 n_g L}, \tag{2}$$

with n_g being the mode group index and L the cavity length. Considering now the simulated mode at the fundamental wavelength, $\lambda = 1550$ nm, which by finite element mode simulations we estimate having a group index of $n_g = 2.19$ and we illustrate in Fig. SF7b, we estimate two cavity lengths of $L_1 = 3.13$ mm and $L_2 = 3.26$ mm (an additional factor of 2 is to be added at the denominator of Eq. 2 as we measure the resonance at the SH wavelength). The test waveguide used for the measurements has a design length of approximately 3 mm, and grating couplers have a length of 100 µm (IR) and 30 µm (visible),

respectively, calculated from the beginning of the taper section to the last grating bar. In total, grating couplers add 130 µm to the straight waveguide section, which corresponds to the difference between the two calculated cavity lengths from the FSRs. This leads us to the conclusion that the interference fringes affecting the SH spectrum originate from two reflections of the fundamental mode: a stronger resonance from reflections at the beginning of the grating couplers tapers, and a weaker reflection from the outermost bars of the two gratings. The fundamental resonances are then inherited by the SH wave which manifests the same behaviour.

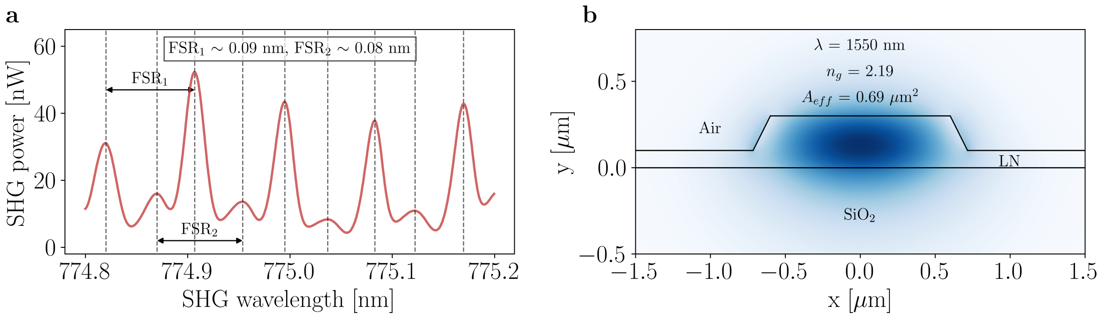

Figure SF7. **Origin of resonance in the second harmonic spectrum.** **a** Detail of the measured second-harmonic spectrum around 775 nm, indicating with vertical dashed lines the two Fabry-Perot resonances and respective free spectral ranges. **b** Simulated optical mode at the second-harmonic fundamental wavelength, featuring a group index of $n_g = 2.19$ and an effective mode area of $A_{eff} = 0.69\,\mu m^2$.

SM2: STATE DESCRIPTION AND DEVICE CALIBRATION

After propagation through the two analysis interferometers and before detection, the overall state between signal and idler channels can be described as

$$\begin{aligned}|\tilde{\psi}(\varphi)\rangle = \frac{1}{2\sqrt{2}}[&|E_sE_i\rangle + \\ &+ e^{i\varphi_i}|E_sT_i\rangle + e^{i\varphi_s}|T_sE_i\rangle + \\ &+ \left(e^{i(\varphi_s+\varphi_i)} + e^{i\varphi_p}\right)|T_sT_i\rangle + \\ &+ e^{i(\varphi_i+\varphi_p)}|L_sT_i\rangle + e^{i(\varphi_s+\varphi_p)}|T_sL_i\rangle + \\ &+ e^{i(\varphi_s+\varphi_i+\varphi_p)}|L_sL_i\rangle],\end{aligned} \tag{3}$$

where subscripts indicating signal and idler channels are explicitly indicated. We would like to stress that states pertaining to second and fourth lines of the equation arise only because photons are probabilistically split at the Franson interferometers inputs and do not carry useful information. This is due to the fact that they can be associated to distinguishable paths being taken by photons, which are projected onto opposite bases. Namely, $|E_sT_i\rangle + |T_sE_i\rangle$ states arise from early photons taking opposite paths on the two twin interferometers, and similarly for the $|L_sT_i\rangle + |T_sL_i\rangle$ states originating from late photons. Recording triple coincidence events (signal, idler and pump) allows to observe all the possible states if displayed in a two-dimensional histogram, which is reported in Fig. SF8a in a slightly modified version of Fig. 3a in the main manuscript. Specifically, diagonal lines are superimposed to the two-dimensional histograms to better illustrate the correspondence between it and its collapsed view in panel Fig. SF8b. By summing counts falling within the ellipses intercepted by each line, from bottom-left to top-right (excluding the black dashed regions), one can build the triple coincidence measurement of Fig. SF8b, which features five peaks [3] and reveals how second and third columns do not carry useful information as they are formed by a superposition of distinguishable, non-interfering states.

Calibration of quantum interference is performed, as described in the main text, by sweeping the electrical power applied to each delay-line phase shifter and monitoring its oscillation as a function of the relative phase. The probability of detecting the state $|T_sT_i\rangle$ can be easily obtained from Eq. 3 by projecting the overall state, namely

$$P = |\langle T_sT_i|\tilde{\psi}(\varphi)\rangle|^2 = \frac{1+\cos(\varphi_s+\varphi_i-\varphi_p)}{4}. \tag{4}$$

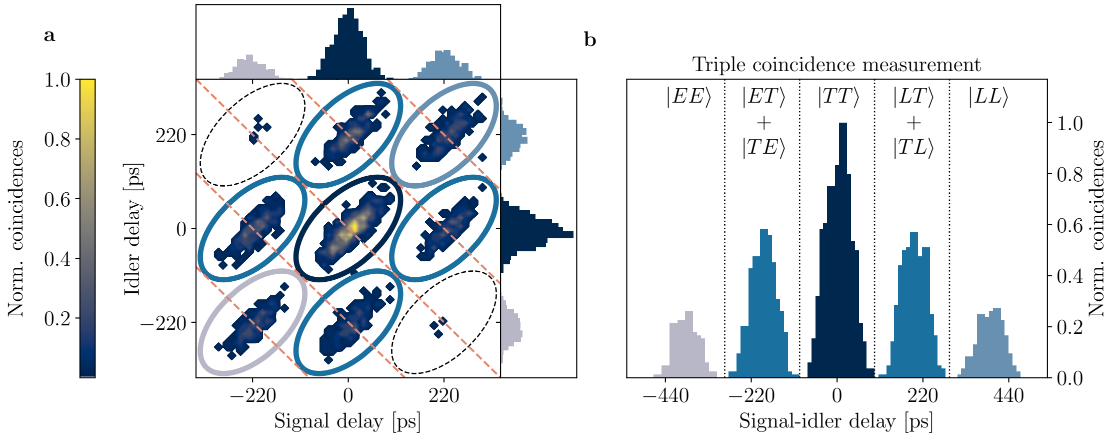

Figure SF8. **Extended Figure 3: Triple coincidence measurement.** **a** Diagonal lines are superimposed to the two-dimensional histogram in order to better highlight the correspondence between each state and its representation in the collapsed view of panel **b**.

Figure SF9a shows raw calibration data as obtained during the measurement, with top and side inset showing horizontal and vertical slices along the centre of the map, respectively. The solid line illustrates the result, on each channel, of a two-dimensional fit to the data based on Eq. 4, with the complete fitted function being displayed in Fig. SF9b. The dashed rectangular region superimposed to the map illustrates the path to follow in order to span the two qubits Bloch spheres equator, and the diagonal arrow indicates the offset introduced by the relative phase between pump pulses, φ_p.

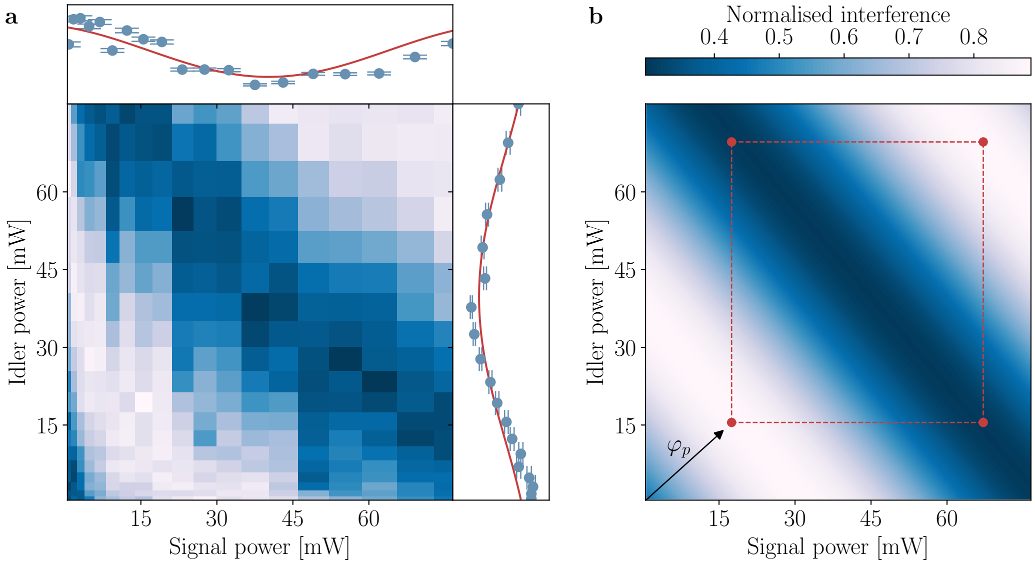

Figure SF9. **Extended Figure 2: Quantum interference calibration map.** **a** Raw data, sliced along horizontal and vertical lines at the centre of the map being displayed in the top/side insets along with slices of the two dimensional fit being performed on the data to obtain the device calibration. **b** Two-dimensional fit, with a dashed rectangle superimposed to the map in order to indicate the path to be followed to span the two qubits Bloch spheres equator.

SM3: EFFECTS IMPACTING VISIBILITY

Three main effects can be identified to be source of interferometric visibility loss: unbalanced optical losses among the interferometers arms, chromatic dispersion due to the broad photon bandwidth and excessively high photon-pair generation probability per pulse.

Optical losses are balanced by using variable optical attenuators, thus by tuning the electrical power, hence the splitting ratio of the Mach-Zehnder interferometers on the short interferometer arm. An example operation of the VOA is displayed in Fig. SF10. We couple a femtosecond telecom laser to the devices, pulses are split by interferometer and we use a fast photodiode and oscilloscope to record the intensity traces of the resulting pulses. By tuning the applied electrical power, part of the optical power is damped into the second VOA output port until the two pulses have equal amplitude. From this measurement we estimate an additional propagation loss of approximately 1 dB due to the long spiral waveguide imparting the delay. Ripples around the pulses traces originate from the impulse response of the photodiode.

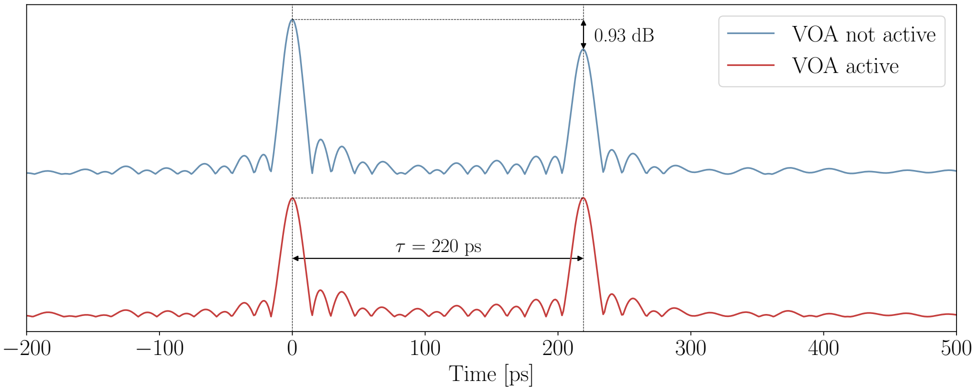

Figure SF10. **Variable optical attenuator operation.** The top trace displays pulse pairs at the interferometer output when the VOA is not biased, while the bottom trace illustrates two balanced pulses due to the tuned splitting ratio.

Concerning chromatic dispersion, we design our circuits by carefully considering anisotropy of the crystal in order to predict the delay. We simulate an optical mode as it propagates at an angle relative to the two crystal axes. We build an effective index ellipse using the simulated mode properties and are thus able to track the mode as it propagates along arbitrarily oriented trajectories on the crystal plane. Figure SF11 shows, on the left panels, an

example simulation of delay line (a) and the resulting broadened pulse (b) of $\Delta\lambda \sim 18.5$ nm bandwidth (red) after propagation across the long interferometer arm. The blue trace is the calculated interferogram after recombination of pulse pairs having travelled across the circuit, thus having experienced different amounts of dispersion along the short and long interferometer arms. Figure SF11c shows pulse propagation measurements we conducted in the early stages of the work to assess the quality of our design procedure. We fabricated multiple variants of the interferometer, with increasing delay. The reported traces are measurements of pulse pairs being generated by the unbalanced interferometer, with designed increments of 20 ps. The pulse traces are not representative of the actual optical pulses as they are convolved with the impulse response of the fast photodiode used during measurements, only delays are to be considered. VOAs were not used during such tests.

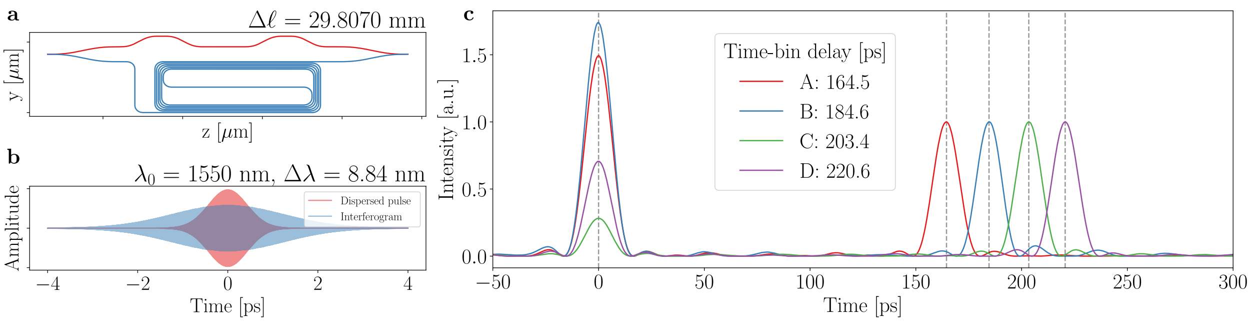

Figure SF11. **Circuit design procedure and test.** **a** Simulated circuit for pulse propagation, short and long interferometer arms are distinguished in red and blue, respectively. **b** Simulated propagated broadened pulse (red trace) across the long interferometer arm and resulting interferogram (blue trace) for an input pulse at 1550 nm with a bandwidth of $\Delta\lambda \sim 8.8$ nm. **c** Measured pulse pairs obtained from test interferometers with increasingly longer delays: designed incremental delay of 20 ps.

Figure SF12 displays the simulated visibility values as a function of photon bandwidth (Fig. SF12a) and pumping strength (Fig. SF12b). In Fig. SF12a we indicate with dashed lines the visibility threshold for assessing the presence of entanglement, $V = 70.7\,\%$, which corresponds to a photon bandwidth of approximately 10.5 nm, the maximum visibility that we can observe given the full-width at half-maximum of our bandpass filter, and the expected visibility when a narrower filter with 5 nm bandwidth would be used. This shows that visibility could be greatly enhanced by further narrowing down the post-selected photon

bandwidth, yet this would also cause a substantial decrease in photon counts. In future work we will investigate narrower bandpass filters and higher pump powers to achieve higher visibility and count-rates. The broad experimental bandwidth could nonetheless be exploited to enhance information capacity by wavelength-multiplexing the experiment, which is impossible with silicon-based platforms. This result shows that our measurement approaches the dispersion limit as we observe 78.1±2.0 % against the maximum expected of 79.4 %. Deviations from the maximum theoretical visibility are attributed to effects such as non-perfectly balanced optical losses in the devices and detector dark counts.

We extract the interferometric visibility as a function of the pumping strength by simulating propagation through the interferometer of states of progressively lower purity. We sweep the probabilities of generating zero- or two-pair states and observe how these affect the measurable visibility by assuming monochromatic fields and lossless propagation. We then model SPDC as a squeezing process of the vacuum field (cfr. [4], Chapters 2 and 16) by applying the squeezing operator

$$\hat{S}(\xi) = \exp\left(\frac{1}{2}\xi^* \cdot (\hat{a})^2 - \frac{1}{2}\xi^* \cdot \left(\hat{a}^\dagger\right)^2\right) \tag{5}$$

to the vacuum state $|0\rangle$. The complex squeezing parameter is expressed as $\xi = se^{i\theta}$, with $s \geq 0$ being related to the second order nonlinear conversion process via $s \propto \chi^{(2)} A_p L$. A_p is the un-depleted pump field amplitude and L the nonlinear interaction length (cfr. [5], Chapter 9). In this sense, the parameter s is equivalent to the pumping field strength. The resulting squeezed state can be written in the Fock basis as

$$|\xi\rangle = \sqrt{\operatorname{sech}(s)} \sum_{n=0}^{\infty} \frac{\sqrt{(2n)!}}{n!} \left[-\frac{1}{2} e^{i\theta} \tanh(s)\right]^n |2n\rangle\,, \tag{6}$$

where n indicates the number of photon pairs generated during the down-conversion process (cfr. [5], Chapter 7). The probability of generating n photon pairs as a function of the squeezing parameter is then

$$P(s,n) = |\langle 2n|\,\xi\rangle|^2 = \operatorname{sech}(s) \frac{(2n)!}{2^n (n!)^2} \tanh^{2n}(s). \tag{7}$$

In our experiment, we pump the nonlinear crystal with pulse pairs of known down-conversion probability per pulse, and we can model the entangled state generation by considering the probabilities of generating zero (p_0), one (p_1) or two (p_2) photon pairs per each pair of pulses

as

$$
\begin{aligned}
p_0(s) &= P(s,0)^2 \\
p_1(s) &= 2P(s,0)P(s,1) \\
p_2(s) &= \underbrace{2P(s,0)P(s,2)}_{\text{two pairs from either pulse}} + \underbrace{P(s,1)^2}_{\text{one pair from each pulse}} ,
\end{aligned} \tag{8}
$$

where $P(s,1) = p$ is the measured probability of down-conversion from a pulse, and events with $n > 2$ can be neglected at reasonably low pump power. Figure SF12b reports the calculated visibility, under the above conditions, as a function of the real part of the squeezing parameter, s. We highlight with dashed lines the threshold for CHSH inequality violation and the value of s corresponding to our experimental conditions, thus confirming that the observed visibility is solely limited by chromatic dispersion of broadband photons in the waveguide as effects originating from un-pure state generation can be neglected. Definition of the visibility at $s = 0$ has no physical meaning as interference cannot be observed in absence of generated photons.

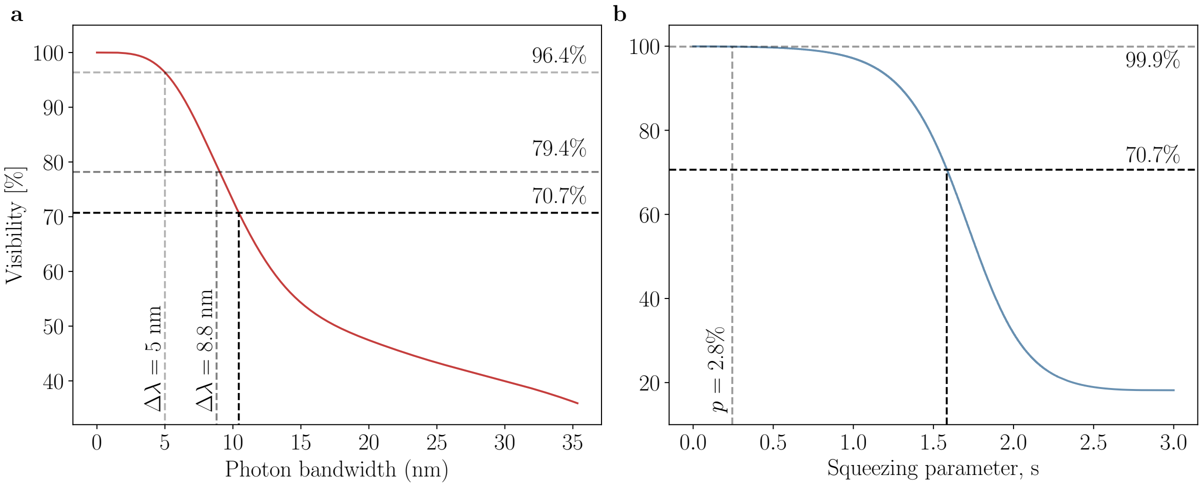

Figure SF12. **Interfering state visibility. a** Visibility as a function of signal and idler photon bandwidth. **b** Visibility as a function of the squeezing parameter for the SPDC process. Operating point, and threshold for detecting entanglement are marked with dashed lines, confirming that our experiment is conducted at the limit of chromatic dispersion.

[1] D. N. Klyshko, Utilization of vacuum fluctuations as an optical brightness standard, Soviet Journal of Quantum Electronics **7**, 591 (1977).

[2] I. Marcikic, H. de Riedmatten, W. Tittel, V. Scarani, H. Zbinden, and N. Gisin, Time-bin entangled qubits for quantum communication created by femtosecond pulses, Physical Review A **66**, 062308 (2002).

[3] H. Jayakumar, A. Predojević, T. Kauten, T. Huber, G. S. Solomon, and G. Weihs, Time-bin entangled photons from a quantum dot, Nature Communications **5**, 4251 (2014).

[4] M. O. Scully and M. S. Zubairy, *Quantum Optics* (Cambridge University Press, Cambridge, 1997).

[5] C. Gerry and P. Knight, *Introductory Quantum Optics* (Cambridge University Press, Cambridge, 2004).
